# Michigan State Veterinary Medical Association

**Published:** 1897-03

**Authors:** 


### MICHIGAN STATE VETERINARY MEDICAL ASSOCIATION.

### DEATH OF DR. J. D. RUTHERFORD, M.D., V.S., OF THE VETERINARY DEPARTMENT, DETROIT COLLEGE OF MEDICINE.

WHEREAS, It has pleased Divine Providence to call from us our highly esteemed friend and brother, Dr. J. D. Rutherford, who so recently enjoyed the various blessings of this world and who was so rapidly rising in the ranks of his chosen profession, at the same time gathering about him a multitude of friends, who will ever regret his departure; therefore, be it

*Resolved*, That we, the members of this Association in convention assembled to-day, extend to his bereaved family our most earnest and heartfelt sympathies in this the hour of their affliction, and at the same time do express our great sorrow that one so valuable in our ranks should be taken from among us. But we console ourselves with the thought and belief that he has gone to a world where all is peace and happiness.

*Resolved*, That a copy of these resolutions be forwarded to his wife and family, as well as the veterinary journals of this country, and that the same be spread upon the records of this Association.

E. A. A. GRANGE, V.S.,
J. A. DELL, V.S.,
G. W. DUNPHY, V.S.,
Committee on Resolutions.

LANSING, MICH., February 2, 1897.

At a meeting of this Association, held in the city of Lansing, Mich., February 2 and 3, 1897, it was unanimously resolved that

WHEREAS, The passage of the bill now pending in Congress, directed toward the abolition of vivisection in the District of Columbia, would perilously impair the scientific experiments of the Bureau of Animal Industry, in which we as members of the veterinary profession are deeply interested; and

WHEREAS, The live-stock interests of this nation represent a large amount of invested capital, which for its successful operation looks for guidance on many vital points to experiments and scientific researches conducted by the Experimental Department of said Bureau of Animal Industry; therefore, be it

*Resolved*, That this Association express its disapproval of such a bill becoming a law; and be it further

*Resolved*, That the several Representatives of the State of Michigan in Congress and the several United States Senators for Michigan be furnished with copies of these resolutions and be hereby requested to vote against such bill and use such influence as they may deem proper to insure its defeat.

J. HAWKINS, V. S.,
D. CUMMINGS, V.S.,
H. M. GOHN, V.S.,
Committee on Resolutions.

